# Correction: The calcium-binding protein S100A1 binds to titin’s N2A insertion sequence in a pH-dependent manner

**DOI:** 10.1085/jgp.20231347210212025C

**Published:** 2025-10-28

**Authors:** Sabrina I. Apel, Emily Schaffter, Nicholas Melisi, Matthew J. Gage

Vol. 157, No. 1 | https://doi.org/10.1085/jgp.202313472 | December 31, 2024

The authors regret that, during manuscript submission, Fig. S3 was also submitted as Fig. S2, resulting in duplicate figures. The published source data file for Fig. S2 is correct as is. The correct Fig. S2 is provided here.

In addition, during the production process, some legend text was mistakenly associated with the wrong figure for Figs. S1, S2, and S3. The corrected legends appear here.

The errors appear in PDFs downloaded before October 24, 2025.

**Figure 1.**
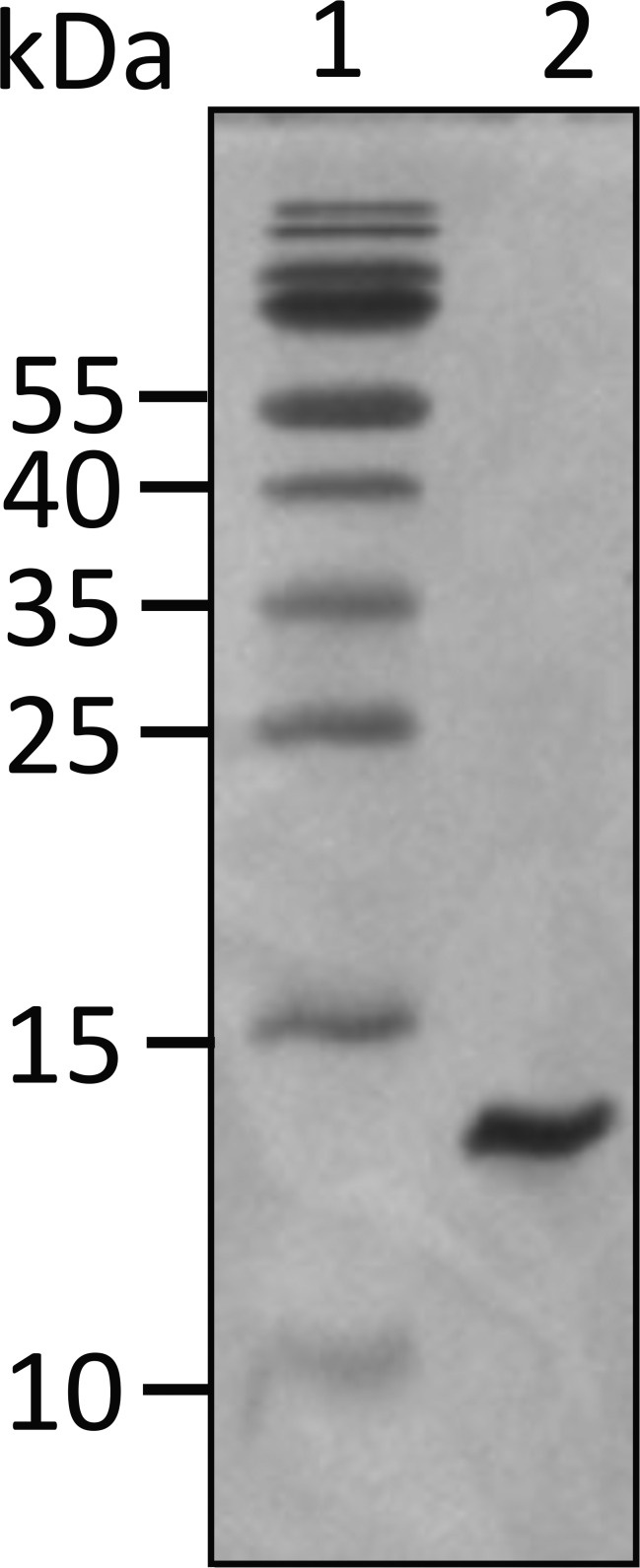
**16% SDS-PAGE gel for UN2A.** The titin gene sequence of the UN2A region was commercially synthesized by GeneArt (Invitrogen) and encompasses amino acids 8539 to 8655 in the mouse titin sequence (GenBank accession no. NM_011652.3), which shares 95% identity with the human UN2A sequence (111 out of the 117 amino acids are identical). Sequence for UN2A: DERKKQEKIEGDLRAMLKKTPALKKGSGEEEEIDIMELLKNVDPKEYEKYARMYGITDFRGLLQAFELLK QSQEEETHRLEIEELEKSERDEKEFEELVAFIQQRLTQTEPVTLIKD. Source data are available for this figure: SourceData FS1.

**Figure:**
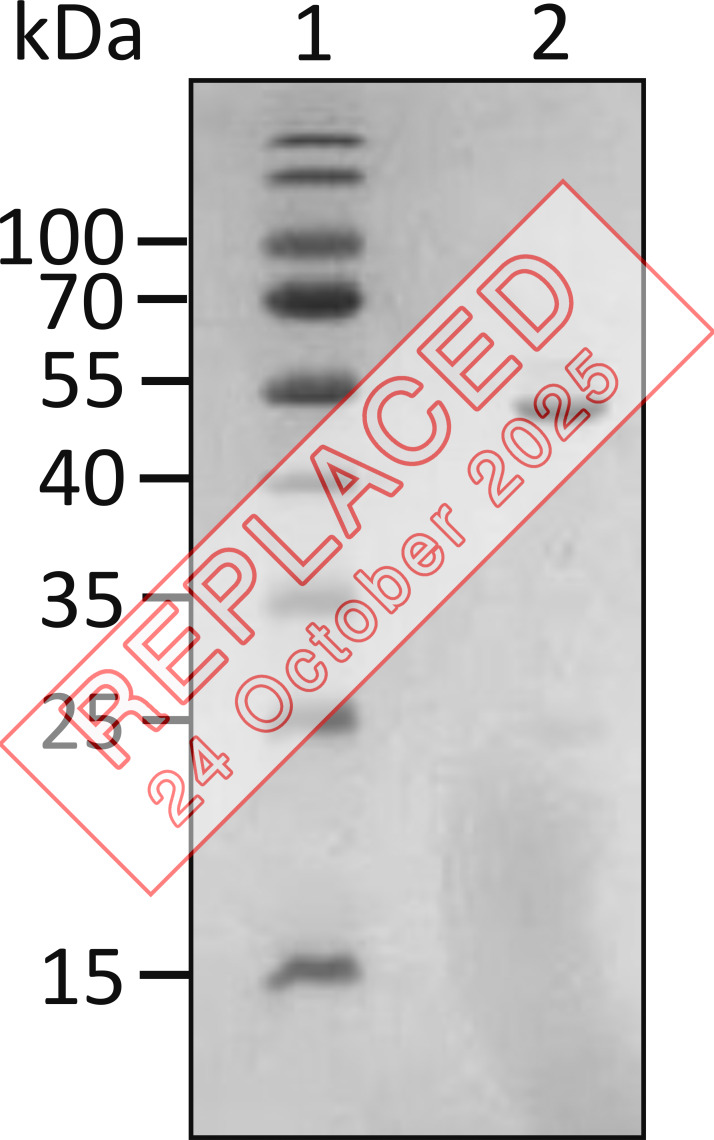


**Figure S2.**
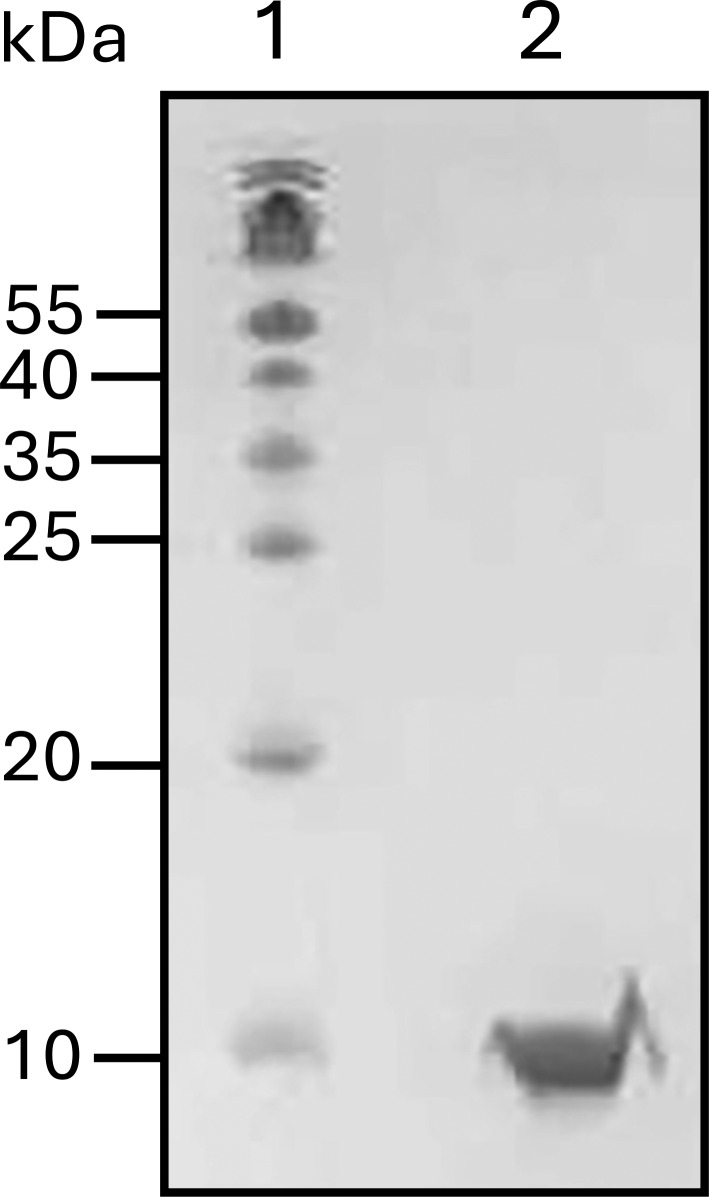
**16% SDS-PAGE gel for S100A1.** The mouse S100A1 gene sequence was graciously provided by David J. Weber’s lab from the University of Maryland. (GenBank accession no. NM_011309.3) Sequence for S100A1: MGSELESAMETLINVFHAHSGQEGDKYKLSKKELKDLLQTELSGFLDVQKDADAVDKVMKELDENGDGEVDFKEYVVLVAALTVACNNFFWETS. Source data are available for this figure: SourceData FS2.

**Figure S3.**
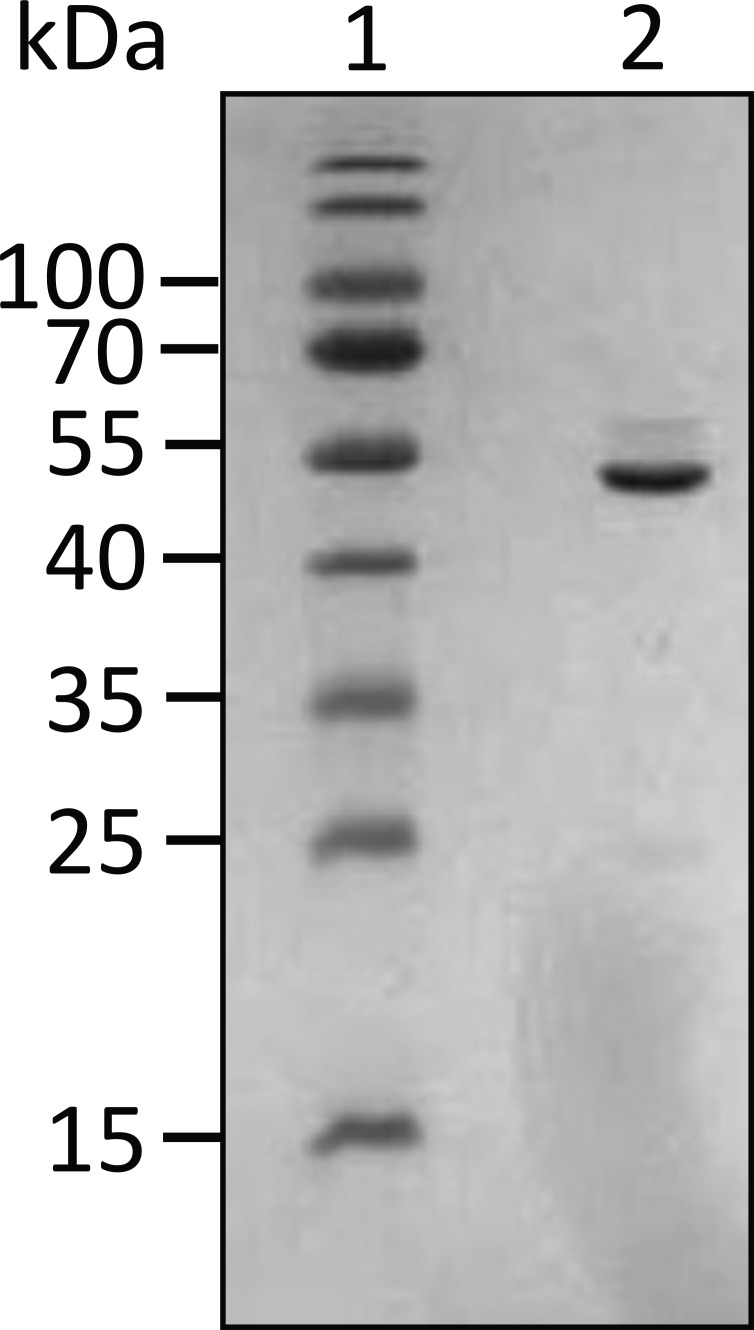
**12% SDS-PAGE gel for UN2A-FRET.** The UN2A region was PCR amplified as described in Tiffany et al. (2017). The resulting PCR product was digested using *NdeI *and *XhoI *and ligated into the CFP-YFP containing vector described by Ohashi et al. (2007). Sequence for UN2A-FRET:HHHHHHMVSKGEELFTGVVPILVELDGDVNGHKFSVSGEGEGDATYGKLTLKLLCTTGKLPVPWPTLVTTLGYGVQCFARYPDHMKQHDFFKSAMPEGYVQERTIFFKDDGNYKTRAEVKFEGDTLVNRIELKGIDFKEDGNILGHKLEYNYNSHNVYITADKQKNGIKANFKIRHNIEDGGVQLADHYQQNTPIGDGPVLLPDNHYLSYQSALFK DPNEKRDHMVLLEFLTAAGITEGMNELYK DERKKQEKIEGDLRAMLKKTPALKKGSGEEEEIDIMELLKNVDPKEYEKYARMYGITDFRGLLQAFELLKQSQEEETHRLEIEELEKSERDEKEFEELVAFIQQRLTQTEPVTLIKDMVSKGEELFGGIVPILVELEGDVNGHKFSVSGEGEGDATYGKLTLKFICTTGKLPVPWPTLVTTLTWGVQCFSRYPDHMKQHDFFKSVMPEGYVQERTIFFKDDGNYKTRAEVKFEGDTLVNRIELKGIDFKEDGNILGHKLEYNYISHNVYITADKQKNGIKANFKARHNITDGSVQLADHYQQNTPIGDGPVILPDNHYLSTQSALSKDPNEKRDHMVLLEFVTAAGITHGMDELYK. Source data are available for this figure: SourceData FS3.
