# Supplementary material for: The calcium-binding protein S100A1 binds to titin’s N2A insertion sequence in a pH-dependent manner
Source: J Gen Physiol. 2024 Dec 31;157(1):e202313472. doi: 10.1085/jgp.202313472 (PMC11687307; doi:10.1085/jgp.202313472)
Supplement: Data S1 — shows RYR and UN2A alignment. [file jgp_202313472_datas1.pdf]

```
UN2A              DERKKQEKIEGDLRAMLKKTPALKKGSGEEEEIDIMELLKNVDPKEYEKYARMYGITDFR
RyR               ------------------------------------------------------------


UN2A              GLLQAFELLKQSQEEETHRLEIEELEKSERDEKEFEELVAFIQQRLTQTEPVTLIKD
RyR               ------------KKAVWHKLLSKN---------------------------------
                              ::   *:*  ::
```
