# Supplementary figures and images for: The calcium-binding protein S100A1 binds to titin’s N2A insertion sequence in a pH-dependent manner
Source: J Gen Physiol. 2024 Dec 31;157(1):e202313472. doi: 10.1085/jgp.202313472 (PMC11687307; doi:10.1085/jgp.202313472)

MW  
(kDa)

35  
25  
15  
10

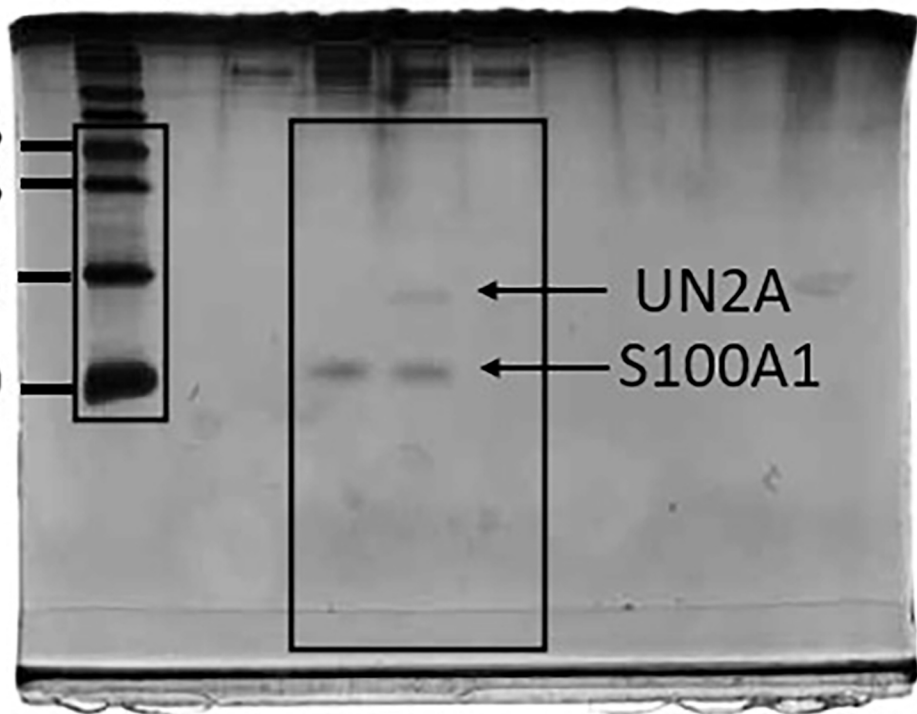

← UN2A

← S100A1

Supplement: SourceData F3 — is the source file for Fig. 3. [file jgp_202313472_sourcedataf3.pdf]

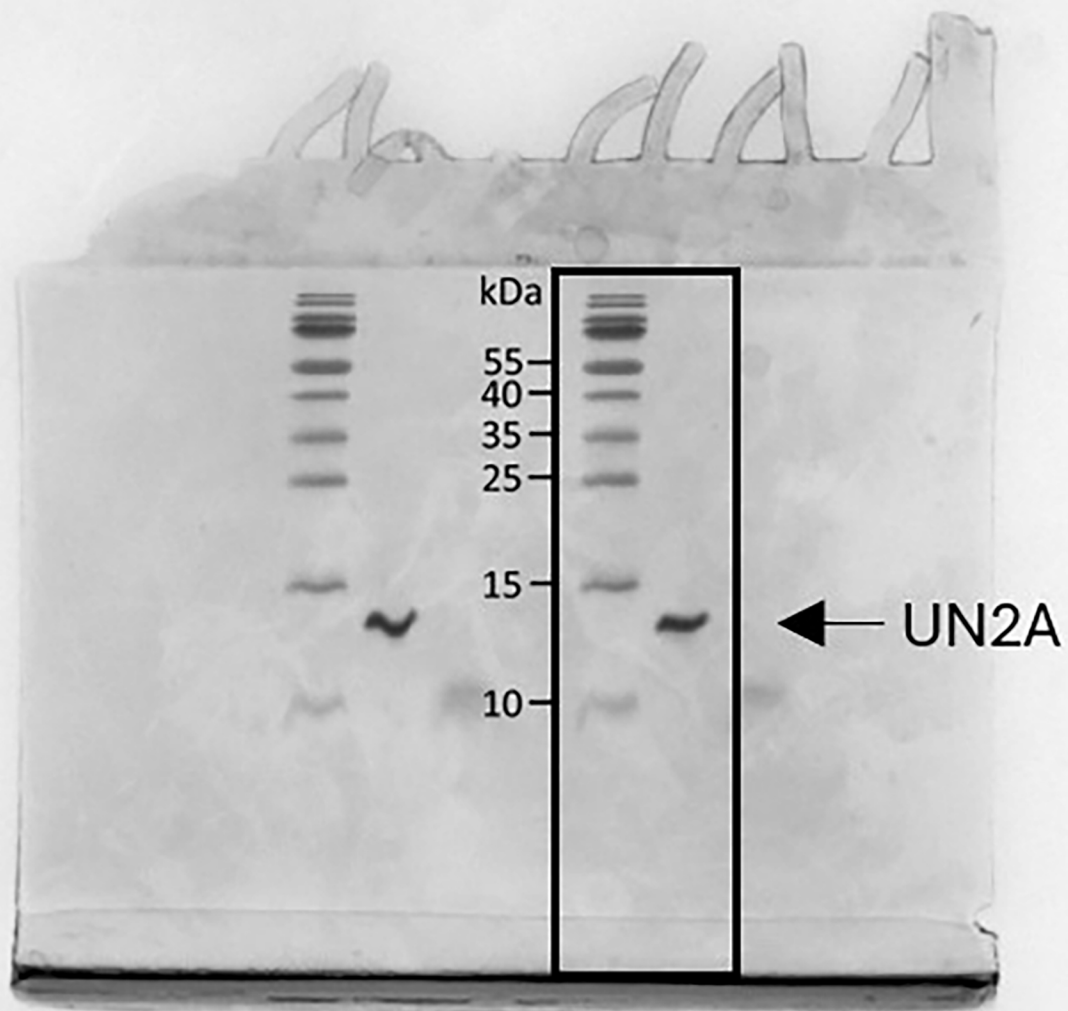

Supplement: SourceData FS1 — is the source file for Fig. S1. [file jgp_202313472_sourcedatafs1.pdf]

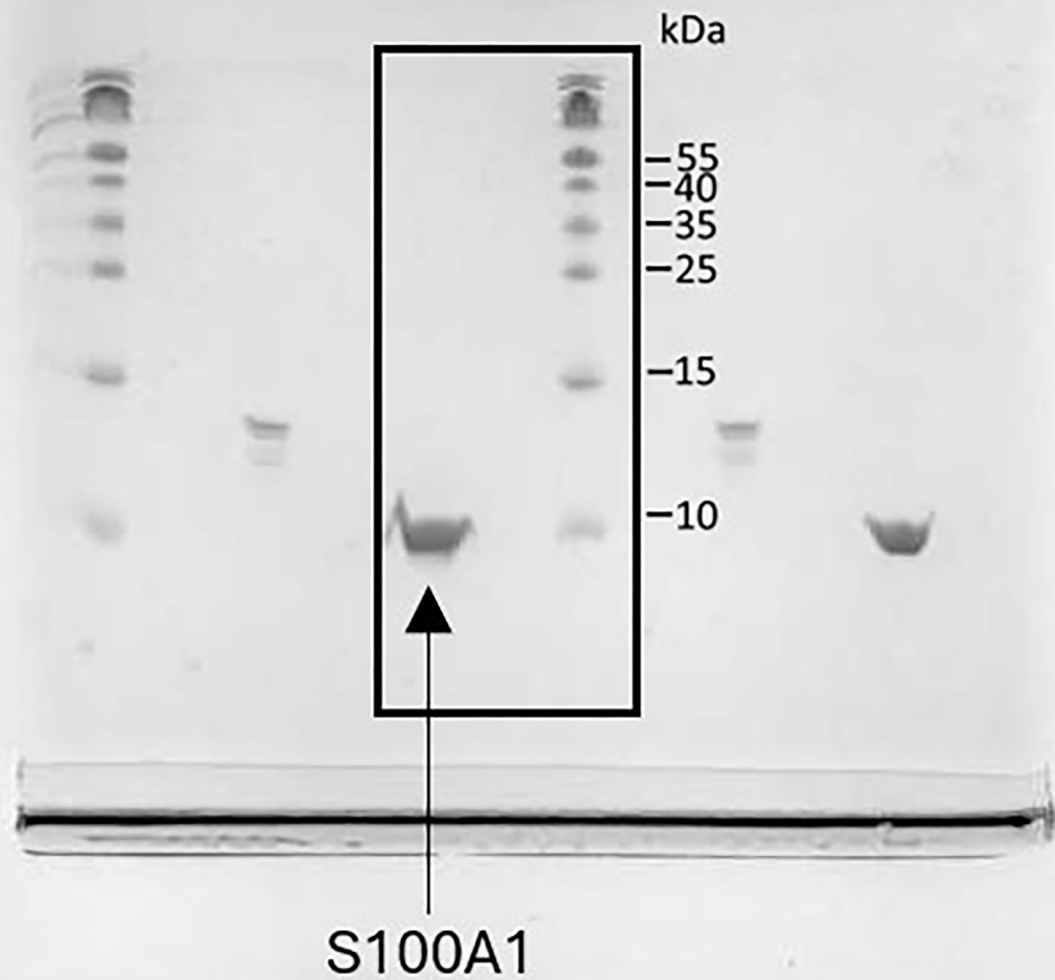

Supplement: SourceData FS2 — is the source file for Fig. S2. [file jgp_202313472_sourcedatafs2.pdf]

kDa

100

70

55

40

35

25

15

← UN2A-FRET

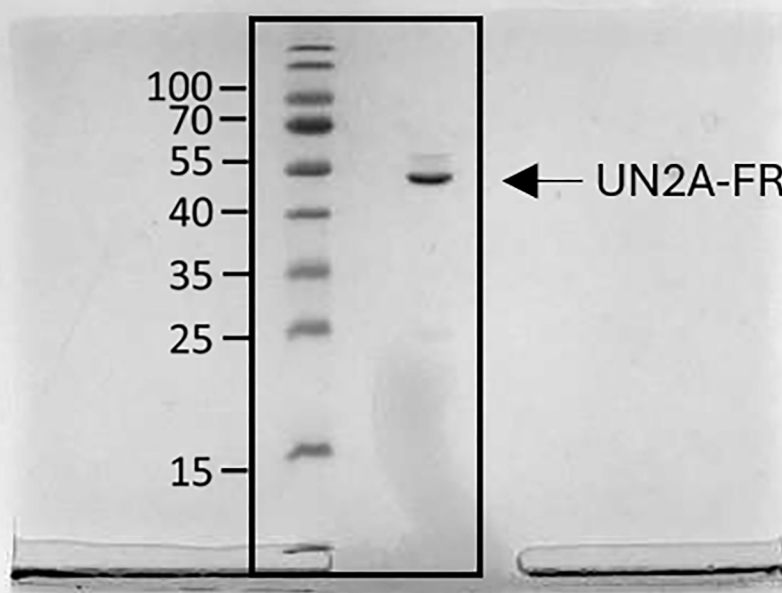

Supplement: SourceData FS3 — is the source file for Fig. S3. [file jgp_202313472_sourcedatafs3.pdf]
